# Combined Solution of Pepsine and Pancreatine*Abstract of a paper by Richard John Kinkead, B. A. and M. T. C. D., in the *Lancet* No. xx. vol. ii. 1870.

**Published:** 1871-04

**Authors:** 


### COMBINED SOLUTION OF PEPSINE AND PANCREATINE.*

The value of pepsine as a remedial agent in cases of indigestion is generally admitted, but experience has proved that it is only in certain forms of indigestion that it is of use.

Food is divided into two classes, nitrogenized and unnitrogenized. The former being digested in the stomach, is acted on by pepsine; the latter, digested in the intestine, escapes its action almost altogether. The only action pepsine, as it appears in the gastric juice, seems to have on fat is to dissolve the albuminous cell-wall, so leaving the fat free to be acted upon by the pancreatic secretion. This suggests a probable cause of indigestion; for if the gastric fluid be deficient in quantity or quality, the albuminous cell-walls of the fat may not be dissolved, the fat is not acted on sufficiently by the pancreatic secretion, and not being emulsified, cannot be taken up by the lacteals. On the other hand, diseases of the pancreas or intestine, by checking the absorption of fat, may cause indigestion incurable by pepsine. This indigestion should be treated by pancreatine, the chief action of the pancreatic secretion being the emulsion of fats.

There being two classes of food to be digested, each in a different portion of the digestive tract, it is evident that the more perfectly one is digested the more easily will the other be. If the stomachic digestion be weak, the fat granules are not set free nor the fibrine dissolved as they should be; the consequence being that the pancreatic secretion cannot do its work properly. If the intestinal digestion be weak, the emulsifying of the fats as they pass from the stomach being imperfectly performed, the food is detained longer in the stomach than is right, the proportion of fat to fabrine is increased, the fat enveloping the nitrogenized food hinders the action of the gastric juice, and acidity and stomachic indigestion are produced. In treating stomachic iudigestion, therefore, it is important to accelerate the digestion of fatty and saccharine portions of the food; and in intestinal to accelerate and perfect the digestion of the albuminoids. There are also cases in which the digestion of both the nitrogenized and unnitrogenized food is at fault.

Impressed with the foregoing ideas, Mr. Edward Long, of Dublin, sent to the author a sample of his solution of pepsine in glycerine, asking him to try it in practice, and give his opinion

*Abstract of a paper by Richard John Kinkead, B. A. and M. T. C. D., in the *Lancet* No. xx. vol. ii. 1870.

upon it. The author, however, thought that a solution of pepsine and pancreatine, combined in suitable proportions, would fulfill the conditions necessary for a perfect digestive; he therefore suggested to Mr. Long the preparation of such a solution. The result of the experiment is given in a letter from Mr. Long to the author, from which we give the following extracts:

"Following up the subject of our conversations some time since, I have been making experiments on pancreatine obtained directly from the fresh pancreas of the calf. The result has been quite what might have been expected from *a priori* reasoning, as you will see from the subjoined statements.

"Some difficulty was experienced in obtaining a solution of pancreatine in an eligible form for administration; but at last I succeeded in producing what as closely as possible represents the digestive fluids found in man. It is composed of pepsine and pancratine in suitable proportions, using for the former a solution of pepsine introduced by me some time ago, and adding the solution of pancreatine as now prepared.

"In the experiments made to test its effects a very curious result was observed. Meat—beef and mutton—digested in pepsine alone was found to be entirely dissolved with the exception of the fat, which floated as a film on the surface, and the film was entirely emulsified when a proper quantity of pancreatine was added, and the usual conditions as to temperature, etc., attended to. This is exactly what we might expect, reasoning from known physiological principles.

"Pepsine in an effectual form has been a great boon; but, as I have shown, it will not digest the oily or fatty aliments; failing thus to supply the system with the substances vitally necessary in strumous diseases. It is obvious how desirable the action of this fluid will be as an addendum to the use of cod-liver oil.

"The pancreatic emulsion has never seemed to me the nicest or most eligible mode of effecting what is desired. It is nauseous to the taste of many, and often keeps badly; the quantity of mutton suet employed, which may be supposed to be all the fatty matter the pancreatine present is capable of emulsifying, is not as much as might be desirable in many cases. In some, suet at all may not be the most suitable form of fat. The fluid I now describe is very palatable, and will keep almost any time. It may be given with any kind of food. My experiments were made with fat mutton-chops and rich beef-steaks, as typical aliments, with most satisfactory results.

"The first experiments, thrice repeated, were made with muriatic acid, water, and the combined solution, to represent the gastric juice and pancreatic secretion. The second, with solution of pepsine alone, with acid and water, followed by the addition of the plain pancreatic solution after an interval of two hours. Both were entirely satisfactory; but the latter were peculiarly interesting in a physiological point of view, as stated above, and tended to show the exact part played by each fluid in the animal economy. But as the administration of two fluids in succession would be troublesome in practice, and be scarcely attended to by patients (at all times averse to trouble,) I have thought it desirable to mix the two in one fluid. This has the advantage being quite agreeable, as liquor of pepsine always is; while the taste of the liquor of pancreatine is entirely concealed by the former. Some medical friends of mine reported most favorably of it, after trial in practice.

"The experiments in the laboratory were as follows:

"No. 1.—Mutton (fat and lean about equal parts,) one ounce; water, one ounce and a half; muriatic acid, fifteen minims; solution of pancreatine and pepsine, one drachm. Digested at 100° for four hours, this was converted into a homogeneous pulp, and then diluted with a little water, presented quite a *chylous* appearance.

"No. 2.—Beef (fat and lean,) an ounce and a half. Treated in same way, with same results, the pulp being much deeper in color.

"Nos. 3 and 4.—I then operated on the same quantities of each, first digesting with pepsine solution alone, as intimated above, then adding the liquor pancreatine—keeping up the heat. In these latter experiments the result seemed more perfect, but, as I have said, the same procedure would be rather inconvenient in practice.

"The results were found to be identical in three successive experiments, at intervals of several weeks."—*Pharm. Jour.*, *London*, *from American Jour. Phar.*

